# A Case of Peritoneal Carcinomatosis After Hyperprogression in Human Papillomavirus-Positive (HPV+) Oropharyngeal Squamous Cell Carcinoma

**DOI:** 10.7759/cureus.62509

**Published:** 2024-06-17

**Authors:** Rafael Bach, Alex Corbera, Anna Sumarroca, Javier Gimeno, Marta Guix

**Affiliations:** 1 Medical Oncology, Hospital del Mar, Barcelona, ESP; 2 Otorhinolaryngology, Hospital del Mar, Barcelona, ESP; 3 Pathology, Hospital del Mar, Barcelona, ESP

**Keywords:** hpv positive, immunotherapy, peritoneal carcinomatosis, hyperprogression, head and neck cancer

## Abstract

Immunotherapy has been shown to provide clinical benefit in selected patients with head and neck squamous cell carcinoma (HNSCC), regardless of human papillomavirus (HPV) infection, and including recurrent/metastatic (R/M) platinum refractory tumors. Hyperprogression is an uncommon negative outcome of treatment with immunotherapy. We present the case of a patient with HPV+ HNSCC who presented hyperprogression after immunotherapy and a rare metastasis location with peritoneal carcinomatosis and subcutaneous nodules. HPV+ HNSCC is related to distant recurrence after a longer interval of time and more diverse metastasis sites compared with HPV- disease. However, the literature on peritoneal metastasis in HNSCC remains limited, with few documented cases. To the best of our knowledge, this is the first case reporting peritoneal carcinomatosis after hyperprogression in HNSCC.

## Introduction

Head and neck squamous cell carcinoma (HNSCC) is the sixth most common cancer. Metastasis occurs in approximately 7% to 20% of HNSCC cases, with the lungs, bones, and liver being the most frequently affected sites [1]. Human papillomavirus (HPV) status plays a crucial role in the clinical behavior of HNSCC, particularly oropharyngeal cancer (OPC). HPV-positive OPC is recognized for its distinct biological behavior compared to HPV-negative disease, including different patterns of metastasis. HPV-positive tumors generally exhibit lower rates of local recurrence and have a better overall prognosis. However, these cases also demonstrate a unique propensity for spreading to more diverse and atypical sites, such as the peritoneum. Furthermore, HPV-positive HNSCC can occasionally manifest unexpectedly aggressive behavior, posing additional challenges for management and treatment [2,3].

## Case presentation

We present the case of a 56-year-old man with no relevant family history of cancer, an ex-smoker with a cumulative rate of 10 packs/year, and a moderate ethanol consumer. His pathological history included chronic hepatitis C treated in 2015 (with undetectable viral load in controls) and bronchiectasis.

In August 2020, he was diagnosed with HPV+ squamous-cell carcinoma of the oropharynx (tonsil), classified as cT2cN1cM0 (stage I), with a combined positive score (CPS) of 10. Initially, he was offered standard treatment with cisplatin 100 mg/m² every three weeks and concomitant radiotherapy (1.6 Gy daily fraction intensity-modulated radiotherapy up to a total dose of 70 Gy). He discontinued chemotherapy after the second cycle (having received 200 mg/m² of cisplatin) due to grade 2 ototoxicity and stopped radiotherapy after 66 Gy by patient decision due to grade 2 radiodermatitis. The patient achieved a complete clinical response.

**Figure 1:**
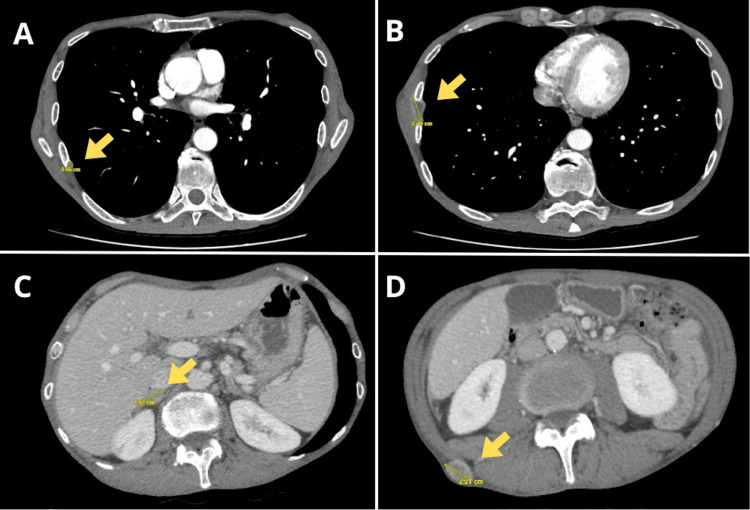
Thoracoabdominal CT, November 2020. Pleural (A, B), adrenal (C), and subcutaneous metastases (D) are observed.

**Figure 2:**
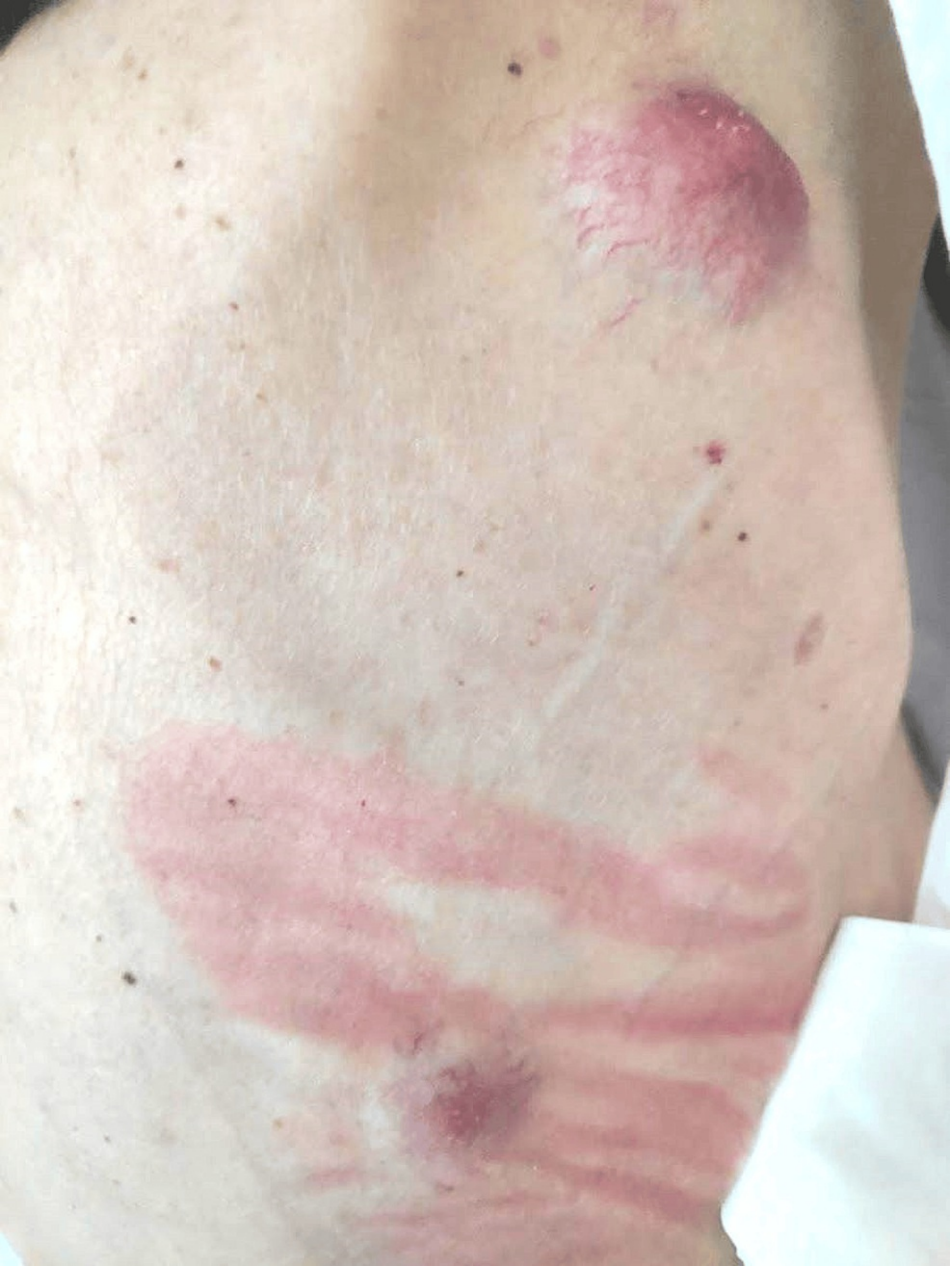
Metastatic subcutaneous nodules.

In November 2020, the patient consulted for the appearance of a subcutaneous nodule on the upper back. Fine-needle aspiration (FNA) cytological study of the subcutaneous nodule revealed HPV+ squamous-cell carcinoma. A subsequent CT scan showed pleural, adrenal, and subcutaneous lesions compatible with metastatic spread (Figures 1, 2).

**Figure 3:**
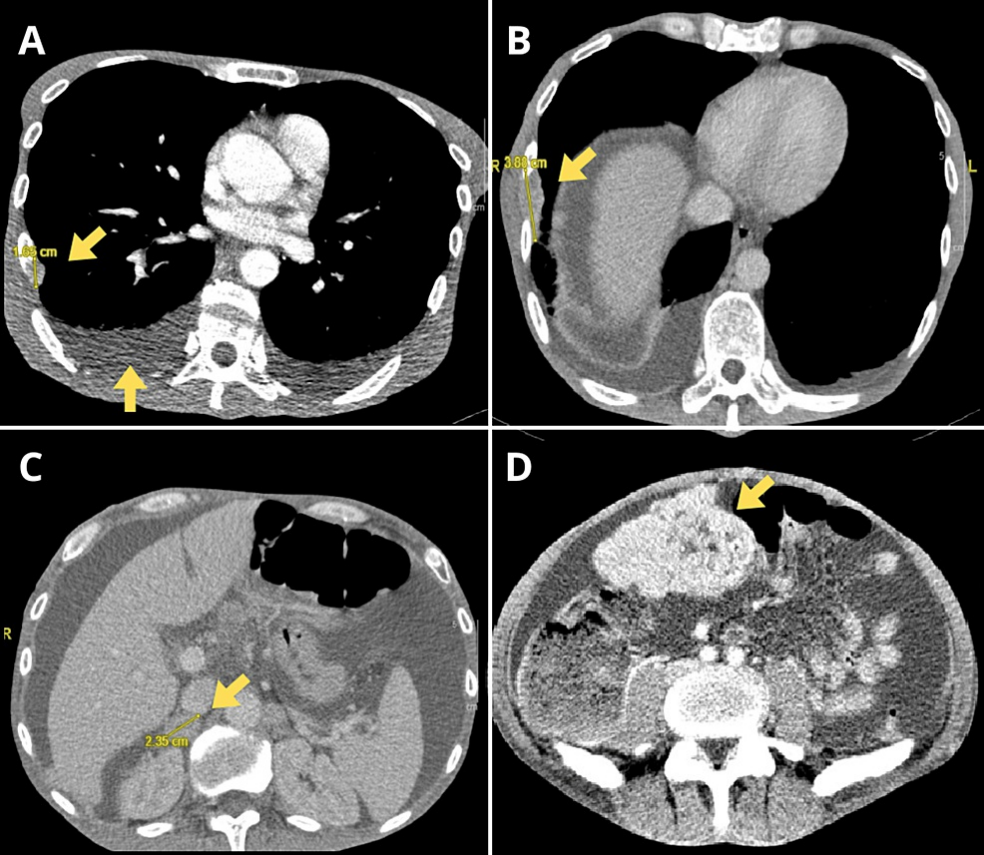
Thoracoabdominal CT, December 2020. Tumor progression, pleural effusion (A, B), ascites (C), and peritoneal carcinomatosis (D) are observed.

Treatment for this early platinum-refractory relapse was initiated with nivolumab 3 mg/kg every two weeks, along with one session of 8 Gy analgesic radiotherapy to one of the nodules. After only two weeks, the patient was admitted to the hospital with a worsening clinical condition, further growth of known subcutaneous nodules, grade 3 anorexia, and grade 3 constipation. On physical examination, the patient had a distended and painful abdomen, absence of peristaltic sounds, and tympanic percussion in the epigastric area. Blood tests showed high neutrophil count (10 x 10^9/L), thrombocytosis (669 x 10^9/L), anemia (11.7 g/dL), decreased albumin (3.1 g/dL), and normal bilirubin, liver enzymes, and coagulation levels. A new CT scan showed peritoneal carcinomatosis with ascites, pleural effusion, multiple bone metastases, growth of previously identified lesions (Figure 3), and new lesions not present on the prior CT scan before starting nivolumab.

**Figure 4:**
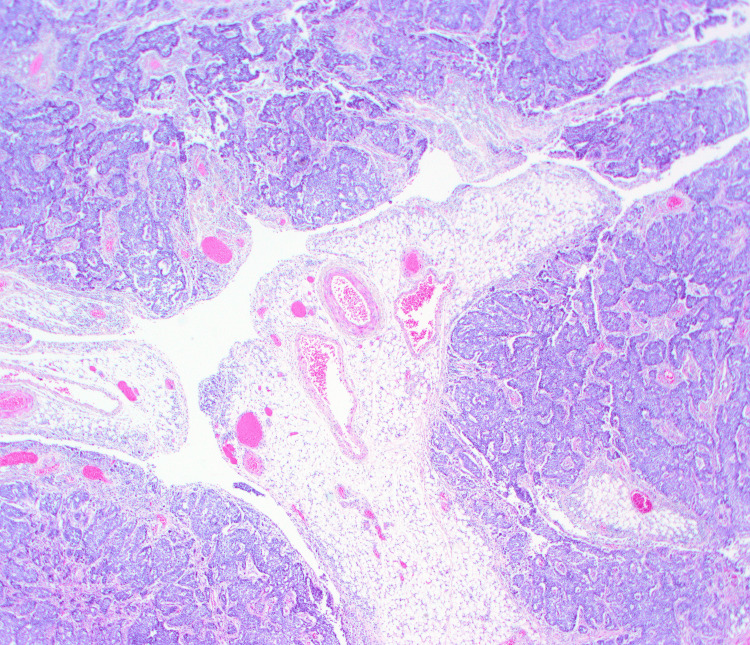
Peritoneal metastasis of HPV+ squamous cell carcinoma with basaloid features (H&E stain, 4X). HPV: Human pappilomavirus, H&E: Hematoxylin and eosin.

Given the symptomatic ascites, paracentesis was performed. The ascitic fluid was serous, and the analysis showed neutrophilic exudate without malignant cells. The patient experienced a rapid decline and died 10 days after hospital admission due to malignant bowel obstruction and respiratory distress. A clinical autopsy, authorized by the family, determined the cause of death as peritoneal carcinomatosis with multiple implants and a 16 cm mesogastric mass causing multiple adhesions and secondary intestinal occlusion. Histological and PCR-based studies of the peritoneal implants confirmed HPV+ squamous-cell carcinoma, consistent with metastasis of the primary tonsillar tumor (Figure 4).

## Discussion

There is scarce evidence in the literature regarding the peritoneal extension of HNSCC, mostly in the context of HPV+ OPC. Huang et al. reported intra-abdominal metastases in eight out of 54 (14.8%) HPV-positive cases, involving the retroperitoneum, mesentery, and iliac lymph nodes. In contrast, no intra-abdominal metastases were observed in HPV-negative OPC [3]. Additionally, Trosman et al. reported three out of 28 cases (10.7%) of intra-abdominal metastases and one out of 28 cases (3.6%) of peritoneal metastasis in HPV-positive OPC [2]. There are also six case reports, one from an HPV+ tumor. The prognosis of these patients was extremely poor, with survival of around one month [4-8]. According to the literature, systemic metastatic spread of the tumor without locoregional regrowth in the head and neck area occurs in up to 5 to 20% of patients. The mean time between diagnosis and distant metastasis is 12 months [1], which was only three months in our clinical case, aligning with the aggressive nature of his cancer.

Immunotherapy was first approved in 2016 for cisplatin-refractory recurrent/metastatic HNSCC [9]. Despite providing clinical benefits to many patients, accelerated tumor growth known as hyperprogression can occasionally occur. This phenomenon has been reported in up to 29% of HNSCC cases [10], with no clear relationship with HPV positivity.

There is no clear consensus on the clinical criteria for hyperprogression. Among the most used criteria are the tumor growth ratio (TGR) and tumor growth kinetics (TGK), both of which have been used in previous studies on HNSCC [10,11]. Both parameters measure the tumor growth acceleration rate between two periods: before and after the start of immunotherapy. To make the comparison, three imaging tests are necessary: ​​before, after, and simultaneous with the start of immunotherapy. Normally, an increase of ≥2 in TGR and TGK is established as a cut-off point. In our case, both parameters are met (TGR ratio = 12.2, TGK ratio = 7). This was also accompanied by clinical deterioration of the patient, who had a performance status (PS) of 0 prior to nivolumab treatment. This fact helps differentiate the episode from pseudo-progression, caused by an inflammatory reaction and followed by a tumor response, usually accompanied by clinical improvement. The management of hyperprogression remains uncertain. Corticosteroids or subsequent lines of treatment with chemotherapy or anti-angiogenic agents have been proposed, but none have demonstrated proven efficacy. Additionally, the deterioration in patients' performance status associated with hyperprogression, as seen in our case report, can limit the feasibility of initiating further treatment options [12,13].

This case highlights the aggressive and atypical presentation of this hyperprogression. In addition to the growth of already-known lesions, new metastases appeared in previously unaffected organs, and peritoneal progression was observed. Ascites, pleural effusion, and the mesogastric mass were not present on previous imaging tests. As this presentation is quite infrequent, we performed a differential diagnosis of the patient's ascites, ruling out common causes such as bacterial peritonitis or decompensated cirrhosis, as the patient did not present fever, altered hepatic enzyme profile, or previously diagnosed cirrhosis. The cytologic exam of the ascitic fluid revealed no malignant cells. However, the clinical autopsy confirmed peritoneal tumor implants of squamous cell carcinoma and the detection of type 16 HPV.

## Conclusions

This case report illustrates a notably aggressive progression of recurrent human papillomavirus-positive (HPV+) head and neck squamous cell carcinoma (HNSCC), culminating in rare peritoneal carcinomatosis following immunotherapy. The rapidity and severity of tumor progression after the initiation of nivolumab treatment exemplify hyperprogression, with a tumor growth ratio (TGR) of 12.2 and a tumor growth kinetics (TGK) ratio of 7. This case highlights an important aspect of HPV+ HNSCC that, despite generally having a better prognosis due to higher responsiveness to treatment, can sometimes exhibit unexpectedly aggressive behavior. This aggressive clinical course raises important questions about potential predictive markers and the intricate interplay between viral oncogenes and the host immune response that might influence tumor behavior. The persistence of HPV positivity in systemic metastases indicates a complex viral-host interaction affecting the tumor's response to immunotherapy.

By highlighting these complexities, this report aims to enhance the medical community's understanding of the potential aggressive behavior of HPV+ HNSCC and emphasizes the importance of considering atypical metastatic pathways in these aggressive cases, adding data to the scarce literature on peritoneal metastasis from HPV+ HNSCC.
